# A longitudinal study on BIO14.6 hamsters with dilated cardiomyopathy: micro-echocardiographic evaluation

**DOI:** 10.1186/1476-7120-9-39

**Published:** 2011-12-08

**Authors:** Maria Paola Belfiore, Daniela Berritto, Francesca Iacobellis, Claudia Rossi, Gerardo Nigro, Ida Luisa Rotundo, Santolo Cozzolino, Salvatore Cappabianca, Antonio Rotondo, Roberto Grassi

**Affiliations:** 1 Institute of Radiology, Second University of Naples (SUN), P.zza Miraglia 2, 80138 Napoli, Italy; 2 Telethon Institute of Genetics and Medicine (TIGEM), Via Pietro Castellino 111, 80131 Napoli, Italy; 3 Biotechnology Center, "A. Cardarelli" Hospital, Via Cardarelli 9, 80131 Napoli Italy

**Keywords:** μ-US, muscular dystrophy, gene therapy, animal model

## Abstract

**Background:**

In recent years, several new technologies for small-animal imaging have been developed. In particular, the use of ultrasound in animal imaging has focused on the investigation of accessible biological structures such as the heart, of which it provides a morphological and functional assessment. The purpose of this study was to investigate the role of micro-ultrasonography (μ-US) in a longitudinal study on BIO14.6 cardiomyopathic hamsters treated with gene therapy.

**Methods:**

Thirty hamsters were divided into three groups (n = 10): Group I, untreated BIO 14.6 hamsters; Group II, BIO 14.6 hamsters treated with gene therapy; Group III, untreated wild type (WT) hamsters. All hamsters underwent serial μ-US sessions and were sacrificed at predetermined time points.

**Results:**

μ-US revealed: in Group I, progressive dilation of the left ventricle with a change in heart morphology from an elliptical to a more spherical shape, altered configuration of the mitral valve and subvalvular apparatus, and severe reduction in ejection fraction; in Group II, mild decrease in contractile function and ejection fraction; in Group III, normal cardiac chamber morphology and function. There was a negative correlation between the percentage of fibrosis observed at histology and the ejection fraction obtained on μ-echocardiography (Spearman r: -0.839; p < 0.001).

**Conclusions:**

Although histological examination remains indispensable for a conclusive diagnosis, high-frequency μ-echocardiography, thanks to the high spatial and contrast resolution, can be considered sufficient for monitoring therapeutic efficacy and/or the progression of dilated cardiomyopathy, providing an alternative tool for repeatable and noninvasive evaluation.

## Introduction

In recent years, several new technologies for small-animal imaging have been developed including micro-radiography (μ-XR), micro-computed tomography (μ-CT), micro-magnetic resonance imaging (μ-MRI), micro-positron emission tomography (μ-PET) and micro-ultrasonography (μ-US). These technologies have allowed for a better evaluation of the efficacy of diagnostic and therapeutic protocols in the field of pre-clinical research. In particular, the use of ultrasound in animal imaging has focused on the investigation of accessible biological structures such as the heart, of which it provides a morphological and functional assessment [1-3].

Muscular dystrophies constitute a heterogeneous group of degenerative diseases characterized by a progressive wasting and weakening of skeletal muscle, of varying severity and distribution [4]. The ethiopathogenesis of these disorders is ascribed to mutations in genes coding for the proteins forming the dystrophin-associated protein complex (DAPC) mutations, which cause loss of integrity of the sarcolemma rendering the muscle fibers more prone to injury [5].

The most frequent types of muscular dystrophy, Duchenne and Becker, are characterized by an X-linked pattern of inheritance, but less common forms also exist having both dominant and recessive autosomal transmission: limb-girdle muscular dystrophies (LGMD). The most severe forms of LGMD (LGMD2D, LGMD2E, LGMD2C and LGMD2F) are due to mutations of alpha-sarcoglycan (SG), beta-SG, gamma-SG and delta-SG, they typically arise in infancy and are often associated with the development of dilated cardiomyopathy [6].

In order to facilitate the understanding of the etiological and pathophysiological mechanisms underlying the different forms of muscular dystrophy and to devise and test new treatment protocols, several animal models have been developed. The BIO 14.6 hamster faithfully reproduces the LGMD2F phenotype seen in humans. In this animal model, the absence of delta-SG from the membrane of skeletal and cardiac muscle results in a secondary deficiency of alpha-, beta-, and gamma-SG. These hamsters exhibit a gradual loss of muscle strength and the progressive development of dilated cardiomyopathy, with a mean lifespan of 11 months, in contrast to the 22 months of the wild type (WT) hamsters [7,8]. The use, in gene therapy, of adeno-associated viruses (AAV) - nonpathogenic, single-stranded DNA viruses of the Parvovirus family - as vectors for cDNA containing the WT delta-SG gene, has been shown to be effective in producing a regression of the pathological phenotype [9,10,6].

This study aimed to define the role of μ-US in the longitudinal study of BIO 14.6 hamsters with dilated cardiomyopathy treated with gene therapy.

## Methods

All procedures performed on the animals received prior approval from our institutional ethics committee. Thirty male hamsters were used; they were maintained on light/dark cycles of 12/12 h and had free access to food and water.

The hamsters were divided into three groups of 10 animals each:

- Group I: untreated BIO 14.6 hamsters;

- Group II: BIO 14.6 hamsters which received an intraperitoneal injection of AAV2/8-CMV-hSCGD (Recombinant AAV vector containing human delta-sarcoglycan cDNA, driven by the cytomegalovirus promoter packaged into AAV2 serotype transcapsidated with AAV8 serotype) after 2 weeks of age and an intrajugular injection of AAV2/1(AAV2 serotype transcapsidated with AAV1 serotype)-CMV-hSCGD after 5 months of age. The AAV were transcapsidated to improve cellular targeting. A different AAV serotype was used in the second injection to prevent immune response against the viral capsid.

- Group III: untreated WT hamsters;

All hamsters were examined with μ-echocardiography and sacrificed with an intrapulmonary injection of 0.2 mg of Tanax at the time points and in the manner shown in Additional file 1.

### One- and two-dimensional echocardiography

Echocardiographic examinations were performed with a VisualSonics Vevo 2100 unit, capable of recording over 750 frames per second and equipped with a high-frequency (40 MHz) transducer.

During each μ-imaging session the hamsters received inhalation anesthesia with isoflurane in oxygen (3-4% for induction and 1.5% for maintenance). Heart rate and body temperature were appropriately monitored and remained constant also throughout the examination. Eutermia was guaranteed by the use of a heating pad. In order to prevent imaging artifacts each hamster was immobilized and its chest hair was removed by applying a calcium thioglycolate depilatory cream. The transducer was fixed on a special arm. An appropriate amount of heated sonographic gel (Aquasonic 100; Parker Laboratories, Inc, Fairfield, NJ) was applied to the shaved skin. The echocardiographic study was performed in two-dimensional (B-mode) and one-dimensional mode (M-mode) in the following projections:

*- Parasternal long axis*

Once the hamster had been prepared for imaging on the animal platform, the transducer was positioned longitudinally to its body, along the right parasternal line, with the index marker pointing to its head and rotated about 35° counterclockwise.

**Figure 1:**
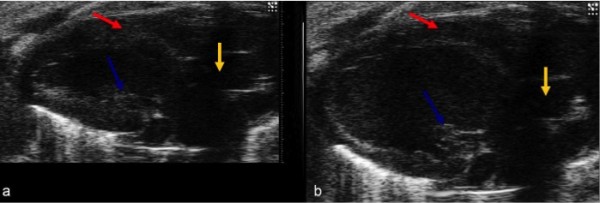
**(a, b) B-mode image of the left ventricle in long-axis view during systole (a) and diastole (b)**. The red arrow shows the interventricular septum, the yellow arrow indicates the aorta and the blue arrow the papillary muscle.

The obtained B-mode image represents a section across the long axis of the left ventricle, which allowed visualization of the aortic root, outflow tract of the left ventricle, delimited anteriorly by the interventricular septum and posteriorly by the anterior flap of the mitral valve (Figure 1a, b).

*- Short parasternal axis*

**Figure 2:**
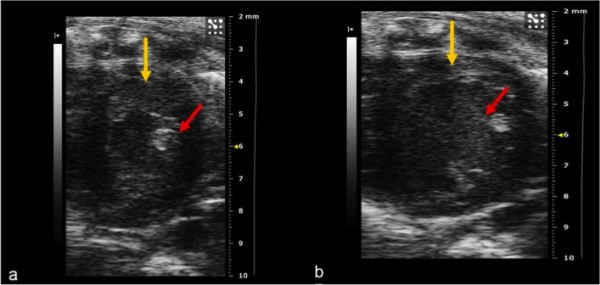
**(a, b) B-mode image of the left ventricle**. Short-axis view during systole (a) and diastole (b). The red arrow shows the papillary muscle, the yellow arrow indicates the thickness of the left ventricle anterior wall. This is the best window for evaluating contractility.

The B-mode parasternal short-axis view was obtained from the long-axis view by rotating the transducer 90° clockwise so that the scan plane was perpendicular to the long-axis of the left ventricle. By moving the transducer downwards, cross-sectional views of the left ventricle were obtained at various levels (mitral, papillary muscles, cardiac apex) along its long axis, which enabled an accurate assessment of cardiac morphometry and global and segmental contractility. On the basis of the B-mode cross-sectional images at the level of the papillary muscles, the following left ventricle measurements were acquired in M-mode: interventricular septum end-diastolic (IVSdt) and end-systolic thickness (IVSst); left ventricular diameter in end-diastole (LVIDd) and end-systole (LVISs); left ventricular posterior wall thickness in diastole and systole (LVPWs, d); left ventricular mass (LVmass); left ventricular volume (LVvol s, d) (Figure 2a, b).

*- Apical*

**Figure 3:**
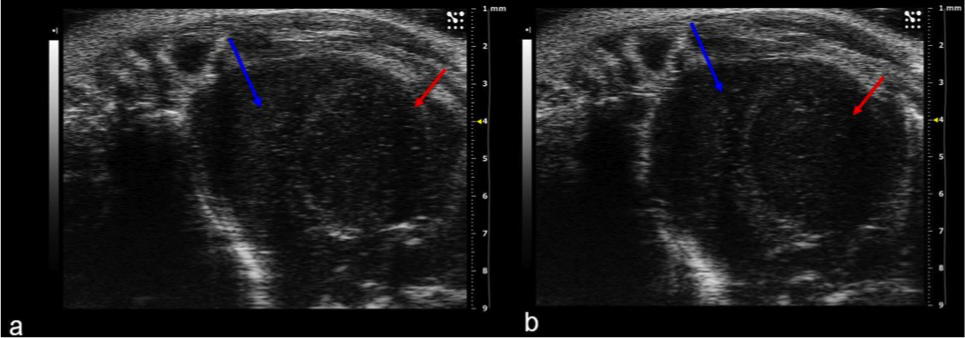
**(a, b) Apical two-chamber view showing right ventricle (red arrow) and left ventricle (blue arrow) during systole (a) and diastole (b)**. The ejection fraction and shortening fraction were calculated with this view.

The bi-dimensional apical view was obtained from the short-axis view, by tilting downwards as far as possible the extremity of the animal platform where the hamster's head was placed and angling the base of the transducer towards the operator. This provided a four-chamber view of the heart. By rotating the transducer clockwise a two-chamber apical view was obtained which is highly important in the study of cardiac contractility (Figure 3a, b).

Left ventricular systolic function was evaluated by calculating the ejection fraction (%EF); this was derived from the left ventricular end-diastolic and end-systolic volumes which were automatically generated by the echocardiographic system software on the basis of the short-axis M-mode measurements - in turn based on the bi-dimensional images - according to the following formula: (LVEDv -LVESv)/LVEDv × 100. Cardiac mass was calculated with the following formula: 0.8 (1.04 ([LVEDd + PWDt + IVSDt]3- [LVEDd]3)) + 0.6.

### Histological examination

After sacrifice of the hamsters at the time points, the cardiac muscle was completely removed and 7-10-micron-thick cryosections were obtained.

The sections were stained with haematoxylin-eosin to quantify the extent of necrotic areas; others were studied after staining with Masson's trichrome stain to assess for the presence of fibrotic areas.

### Statistical analysis

The data are reported as mean ± standard deviation.

The correlation between percentage of fibrosis at histology and ejection fraction at echocardiography was analyzed with the Spearman rank test. Values of p < 0.05 were considered statistically significant.

## Results

### Echocardiography

The results of the echocardiographic study are summarized in Additional file 2.

**Figure 4:**
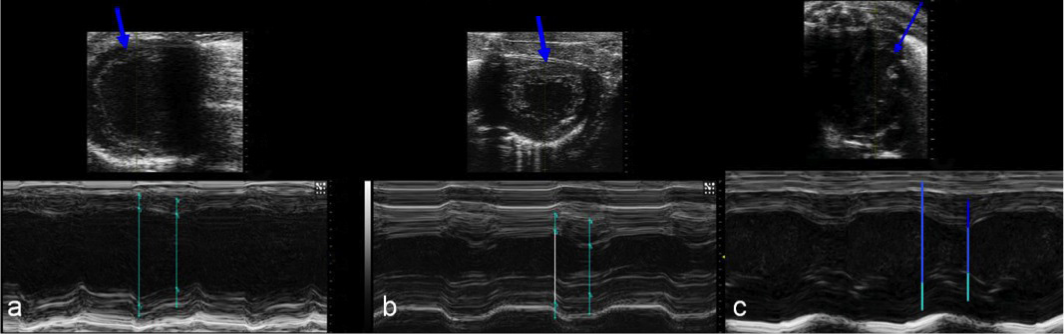
**(a, b, c) Image (a) of group I (untreated) BIO 14.6 hamsters shows reduced myocardial contractility and progression to dilated cardiomyopathy**. The blue arrow indicates thinning of the left ventricle wall. Image (b) of treated BIO 14.6 hamsters reveals slightly reduced myocardial contractility, and preservation of the myocardial wall. Image (c) of wild type hamsters shows preservation of the myocardial wall and no disease state.

The morphological and quantitative data of the echocardiographic study of the hamsters of Group I (untreated BIO 14.6) revealed: dilation of the left ventricle with a change in heart morphology from the physiological elliptical shape to a more spherical shape, altered spatial configuration of the mitral valve and subvalvular apparatus, widening of the left atrium and right atrium and ventricle with severe reduction in ejection fraction at 8 months (Figure 4a, b, c).

In the hamsters of Group II (treated BIO 14.6), echocardiography showed a mild decrease in contractile function and ejection fraction, in presence of a left ventricular wall thickness at the lower limits of normal and mild dilation of the cardiac chambers.

In the hamsters of Group III (untreated WT), echocardiography revealed normal morphology and function of the cardiac chambers.

There was a statistically significant negative correlation between the percentage of fibrosis found at histology and the ejection fraction (Spearman r: - 0.8389; p < 0.001) (see Additional file 3).

### Histological examination

**Figure 5:**
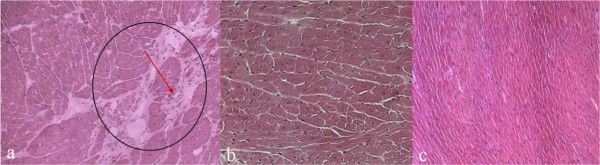
**(a, b, c) Delivery of the delta-sarcoglycan gene through AAV diminishes the percentage of fibrotic areas in hearts**. Haematoxylin-eosin staining on frozen sections: untreated (a), treated (b) and wild type (c). The red arrow indicates the fibrotic areas (a), which are absent from the treated and wild type groups. All frozen sections were analyzed at 7 months of age. Original magnification 20×.

Histological examination performed on the hamsters of Group I revealed degeneration of the myocytes, necrosis and marked fibrosis (see Additional file 4). The hamsters of Group II showed complete absence of areas of necrosis and/or fibrosis, a situation that did not differ significantly from that of the hamsters of Group III (Figure 5a, b, c).

## Discussion

Noninvasive small-animal imaging has taken on an increasing role in preclinical research as to become an independent sector. Today, the availability of advanced imaging techniques constitutes a key factor in the success and timeliness of research thanks to the possibility of conducting longitudinal studies on the same animal [1]. Among these techniques, μ-echocardiography is an inexpensive, repeatable, fast and noninvasive modality and as such it is particularly suited to this type of experimental projects.

Noninvasive imaging helps to render animal experiments more ethically acceptable as it is compliant with the principles of the 3Rs (Replacement, Reduction, Refinement) formulated in 1959, which consider the possibility of replacing [Replacement], where possible, the use and/or sacrifice of the animal with other equally effective methods, reducing [Reduction] the number of experiments, and refining [Refinement] techniques to minimize pain and distress of the animals [11,12]. This study aimed to demonstrate that μ-echocardiography is a powerful tool in support of the 3R policy. Although the modality is well known as a non-invasive, reproducible and inexpensive diagnostic modality, only recently have been developed systems, which allow for a more detailed study of cardiac function in small animals. Thanks to high resolution images (40 MHz) acquisition, with a potential for recording more than 750 frames per second, these systems provide an analysis similar to that obtained in humans and therefore a more objective result of echocardiographic imaging [13,14]. This latter aspect is crucial in order to reduce the number of sacrifices during the experiment, allowing several assessments during follow-up. This should not be regarded as a marginal issue particularly if we consider that, like humans, animals show individual variability in the course of the disease, and further assessment can improve our understanding of the evolution of the disease.

Echocardiography is widely used for the morphological and functional evaluation of the heart [15] as it permits an accurate analysis of cardiac anatomy and haemodynamics facilitating the understanding of the pathophysiological mechanisms underlying the disease being studied. This technique has been able to monitor the effects of gene therapy on the cardiac function of BIO 14.6 hamsters with dilated cardiomyopathy.

Only few ultrasound systems to date have been able to provide high-quality echocardiographic studies on hamsters, because of inadequate image resolution and an inability to process quantitative data. In the present study, despite the high hamsters heart rate, the μ-echocardiographic examination allowed to obtain a precise estimation of heart function parameters thanks to high-frequency transducers (40 MHz).

Indeed, μ-echocardiography is ideal for phenotyping and estimating left ventricular function; as a fast and noninvasive method, it can also be used in animals in suboptimal physical condition.

The lack of the gene coding for delta-SG in these animals entails a loss of sarcolemmal integrity with necrosis of the cardiomyocytes; this promotes the development of an inflammatory response with fibrotic degeneration. By limiting myocardial elasticity, fibrotic replacement reduces contractile function with progressive stretching and thinning of the fibrotic regions, leading to progressive dilation.

The increase in ventricular volume worsens the systolic dysfunction associated with mitral valve insufficiency, leading to low-output heart failure.

Serial echocardiography successfully documented this evolution in the untreated BIO 14.6 hamsters, but not in the treated hamsters. Gene therapy was in fact able to almost completely preserve cardiac function in the dystrophic hamsters, which showed similar morphology, echocardiographic parameters and survival to the wild type hamsters.

In order to confirm the role of this method in monitoring the evolution of cardiomyopathy, samples of myocardial tissue were studied by histological examination. The estimation of injury obtained at microscopic analysis correlated with that of the μ-echocardiographic study.

Our experience showed that μ-US imaging applied to the study of cardiomyopathy in BIO 14.6 hamsters, thanks to the high spatial and contrast resolution, provides an estimation of morphological changes which well correlate with the histological findings, and may help to reduce the animals sacrifice.

Even though histology remains indispensable for a definite diagnosis, echocardiography can be used to monitor the efficacy of treatment and/or the progression of dilated cardiomyopathy and constitutes an alternative tool for a repeatable and noninvasive assessment.

The implementation of power and color Doppler imaging, the use of contrast media and higher-frequency transducers will help to refine the characterization of sonographic patterns of disease also reducing both the time and cost of longitudinal studies.

## Competing interests

The authors declare that they have no competing interests.

## Authors' contributions

MPB, DB and FI performed the research and wrote the manuscript, CR and MC contributed to the conception and design of the study; GN and ILR contributed to the acquisition and interpretation of the data; SC and SC revised the article critically for important intellectual content; AR and RG designed the research and approved the final version for publication. All authors read and approved the final manuscript.

## Supplementary Material

Additional file 1 **Timing and modality of examination of the hamsters**. "Arrow" indicates the timing of gene therapy administration; "heart" represents the time points at which the μ-US examination was performed in each animal; "block" corresponds to the sacrifice of animals to proceed with the histological examination. For each time point, two animals were sacrificed in each group. Point: natural death (Group I: 10,65 ± 0,29 months; Group II: 21,30 ± 0,54 months; Group III 21,85 ± 0,26) the data are reported as mean ± standard deviation. Click here for file

Additional file 2 **Results of the echocardiographic measurements**. IVSs, d - interventricular septum thickness during diastole and systole; LVIDs, d - left ventricular internal diameter during diastole and systole; LVPWs, d - left ventricular posterior wall thickness during diastole and systole; EF% - ejection fraction percentage; LV mass - left ventricular mass; LVvols, d - left ventricular volume during systole and diastole). Click here for file

Additional file 3 **Correlation between percentage of fibrosis and ejection fraction**. The graph shows a significant negative correlation between the percentage of fibrosis and the ejection fraction (Spearman r:-0.8389; p < 0.001) Click here for file

Additional file 4 **Percentage of total fibrosis at histological examination and ejection fraction at micro-echocardiography evaluation**. Click here for file
