# Supplementary figures and images for: A longitudinal study on BIO14.6 hamsters with dilated cardiomyopathy: micro-echocardiographic evaluation
Source: Cardiovasc Ultrasound. 2011 Dec 8;9:39. doi: 10.1186/1476-7120-9-39 (PMC3254069; doi:10.1186/1476-7120-9-39)

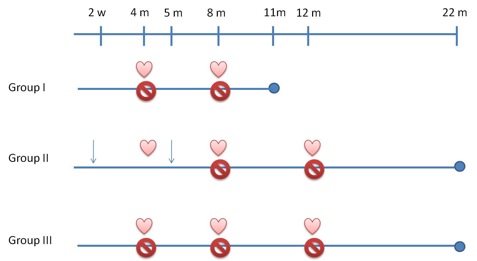

Supplement: Additional file 1 — Timing and modality of examination of the hamsters. "Arrow" indicates the timing of gene therapy administration; "heart" represents the time points at which the μ-US examination was performed in each animal; "block" corresponds to the sacrifice of animals to proceed with the histological examination. For each time point, two animals were sacrificed in each group. Point: natural death (Group I: 10,65 ± 0,29 months; Group II: 21,30 ± 0,54 months; Group III 21,85 ± 0,26) the data are reported as mean ± standard deviation. [file 1476-7120-9-39-S1.JPEG]

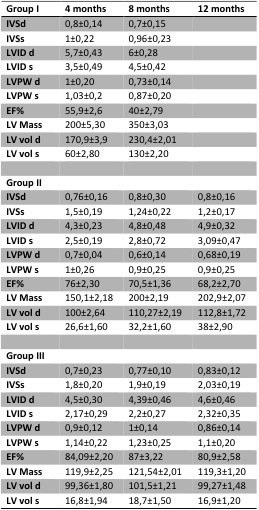

Supplement: Additional file 2 — Results of the echocardiographic measurements. IVSs, d - interventricular septum thickness during diastole and systole; LVIDs, d - left ventricular internal diameter during diastole and systole; LVPWs, d - left ventricular posterior wall thickness during diastole and systole; EF% - ejection fraction percentage; LV mass - left ventricular mass; LVvols, d - left ventricular volume during systole and diastole). [file 1476-7120-9-39-S2.JPEG]

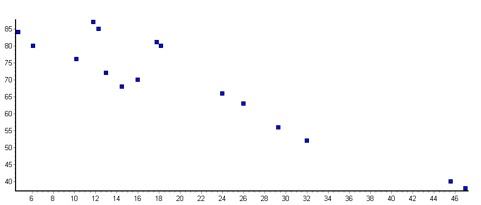

Supplement: Additional file 3 — Correlation between percentage of fibrosis and ejection fraction. The graph shows a significant negative correlation between the percentage of fibrosis and the ejection fraction (Spearman r:-0.8389; p < 0.001) [file 1476-7120-9-39-S3.JPEG]

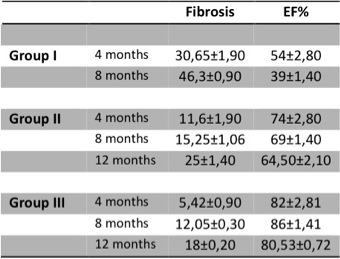

Supplement: Additional file 4 — Percentage of total fibrosis at histological examination and ejection fraction at micro-echocardiography evaluation. [file 1476-7120-9-39-S4.JPEG]
